# Human Papillomavirus Genotype Distribution in Czech Women and Men with Diseases Etiologically Linked to HPV

**DOI:** 10.1371/journal.pone.0021913

**Published:** 2011-07-13

**Authors:** Ruth Tachezy, Jana Smahelova, Martina Salakova, Marc Arbyn, Lukas Rob, Petr Skapa, Tomas Jirasek, Eva Hamsikova

**Affiliations:** 1 Institute of Hematology and Blood Transfusion, Department of Experimental Virology, Prague, Czech Republic; 2 Unit of Cancer Epidemiology, Scientific Institute of Public Health, Brussels, Belgium; 3 Department of Obstetrics and Gynecology, Second Faculty of Medicine, Charles University, Prague, Czech Republic; 4 Department of Pathology and Molecular Medicine, Second Faculty of Medicine, Charles University, Prague, Czech Republic; 5 Department of Pathology, Third Faculty of Medicine, Charles University, Prague, Czech Republic; National Cancer Institute, United States of America

## Abstract

**Background:**

The HPV prevalence and genotype distribution are important for the estimation of the impact of HPV-based cervical cancer screening and HPV vaccination on the incidence of diseases etiologically linked to HPVs. The HPV genotype distribution varies across different geographical regions. Therefore, we investigated the type-specific HPV prevalence in Czech women and men with anogenital diseases.

**Methods:**

We analyzed 157 squamous cell carcinoma samples, 695 precancerous lesion samples and 64 cervical, vulvar and anal condylomata acuminate samples. HPV detection and typing were performed by PCR with GP5+/6+ primers, reverse line blot assay and sequencing.

**Results:**

Thirty different HPV genotypes were detected in our study, HPV 16 being the most prevalent type both in precancerous lesions (45%) and squamous cell carcinomas (59%). In benign lesions, HPV 6 (72%) was the most common type. Altogether, 61% of carcinoma samples and 43% of precancerous lesion samples contained HPV 16 and/or 18. The presence of HPV types related to the vaccinal ones (HPV 31, 45, 33, 52, 58) were detected in 16% of carcinoma samples and 18% of precancerous lesion samples. HPV 16 and/or 18 were present in 76% of cervical cancer samples, 33% of CIN1, 43% CIN2 and 71% of CIN3 samples. HPV types 6 and/or 11 were detected in 84% samples of condylomata acuminate samples.

**Conclusions:**

The prevalence of vaccinal and related HPV types in patients with HPV-associated diseases in the Czech Republic is very high. We may assume that the implementation of routine vaccination against HPV would greatly reduce the burden of HPV-associated diseases in the Czech Republic.

## Introduction

Human papillomaviruses (HPVs) have been established as etiological agents of invasive cervical cancer (CC) [1], [2] and they are the most common viral sexually transmitted infection worldwide. Persistent infection with high-risk (HR) HPVs is necessary for the development of premalignant lesions and/or progression of the disease [3]. Furthermore, HPV has carcinogenic effects at several other anatomical sites in women and men [4]. HPV genotype distribution varies across different populations and geographical regions [5]. Recently, meta-analyses and systematic reviews of HPV type distribution in diseases linked to HPV infections worldwide have been published [6]–[13]. CC is the second most common cancer among women worldwide, with 492,800 incident cases during 2002 [14]. The burden of noncervical anogenital, i.e. anal, vaginal and vulvar, cancers approximates 53,872 cases worldwide annually (i.e. 28,272 anal and 25,600 vaginal and vulvar cancer cases). In the Czech Republic , 990 CC cases, 189 vulvar cancer cases and 121 anal cancer cases occur annually [15].

In spite of the high burden of cervical cancer in Central and Eastern Europe [16], few data are available regarding the prevalence of HPV [17]–[20]. Therefore, our study which collects the available data on Czech patients with a wide variety of HPV-associated diseases will contribute to a better understanding of the HPV type distribution in the Czech Republic. Importantly, it will help in estimating the potential local impact of HPV vaccines on the prevention of HPV-associated diseases in women and men.

## Materials and Methods

### Population studied

Squamous cell cervical carcinoma (SCC) samples as well as precancerous lesion samples from different anatomical locations were selected from the biobank of the National Reference Laboratory for Papillomaviruses in Prague. These samples were collected between 1993 and 2005, stored at −20°C and analyzed in previous studies.

Cervical scrape and biopsy specimens were obtained from women visiting hospital gynecology departments and selected centers of gynecologic-oncology prevention in the Czech Republic [21]. These settings are located in different districts across the Czech Republic and serve wide catchment areas. Therefore, the patients included in our study are representative of the population of the whole of the Czech Republic. Additionally, samples from patients treated for cervical intraepithelial neoplasia garde 1 to 3 (CIN1/2/3) were used. The patientś characteristics and sample processing were published before [22]. The classification of all CIN2/3 and SCC specimens and of the majority of CIN1 (86%) specimens was done by histology as specified before. Overall, 86 SCC specimens (patient mean age 49.7 years; age range 28–87 years), 338 CIN1 specimens (mean age 33.8 years; age range 16–76 years), 111 CIN2 specimens (mean age 34.5 years; age range 20–59 years), and 200 CIN3 specimens (mean age 33.9 years; age range 20–66 years) were selected for the purpose of the present study.

Samples from patients surgically treated in the Department of Obstetrics and Gynecology of the 2^nd^ Faculty of Medicine, Charles University, Prague for squamous cell vulvar carcinoma (VC), vulvar intraepithelial neoplasia (VIN) and vulvar condylomata acuminata (VCA) were also included in the study. The patientś characteristics and histological data were published before [23]. For HPV typing, 49 VC samples (patient mean age 70.7 years; age range 32–95 years), 46 samples from patients with different grades of usual VIN (u-VIN) (patient mean age 52.5 years; age range 29–85 years) and 54 VCA samples (patient mean age 30.6; age range 15–59 years) were available.

Twenty-two samples from patients with squamous cell carcinoma of the anus (AC) (mean age 64.2 years; age range 47–86 years, 18 women & 4 men) and 10 samples of anal condylomata acuminata (ACA) samples (patient mean age 41.4 years; age range 21–69 years, 1 woman & 9 men), were analyzed. Details on the population, sample preparation and pathological classification were published before [24].

Overall, 157 cancer samples from multiple locations, 695 pre-malignant neoplasia samples, and 64 condylomata acuminata samples were included in this study.

### Ethic statement

No informed consent was needed from the patient by the course of law in the Czech Republicbefore 2000. All patients enrolled after the year 2000 signed an informed consent form and the study was approved by the institutional ethics committee [25].

### HPV detection and genotyping

PCR and reverse line blot hybridization (RLB) were used for the detection and genotyping of the HPV DNA in samples [26]. RLB is able to identify 37 different HPV types in a single assay. The HPV detection was performed in a PCR thermocycler PTC 200 (MJ Research, Inc, Waltham, MA, USA) by the PCR assay with primers GP5+ and 5′-end biotin labelled GP6+ primer which amplify the 150 bp fragment of the L1 gene. The PCR was performed for 40 cycles and the biotinylated PCR product was hybridized with the oligonucleotide probes labelled with the 5′-terminal amino-group. These probes were covalently linked to an activated negatively charged Biodyne C membrane. After washing, the membrane was incubated for 60 min at 42°C with peroxidase labelled streptavidin conjugate. For chemiluminescent detection of hybridising DNA, the membrane was incubated in ECL detection liquid (Amersham Biosciences, Uppsala, Sweden) and exposed to LumiFilm (Roche, Indianapolis, IN, USA) for 5 min.

Detected HPV types were classified into low-risk (LR) (HPV 6, 11, 40, 42, 43, 44, 54, 61, 70, 72, 81, and 89), high-risk (HR) (HPV 16, 18, 31, 33, 35, 39, 45, 51, 52, 56, 58, 59, 68,) and probably high-risk (pHR) (HPV 26, 53, 66, 73, 82) types of the genus Alpha that contains the mucosal types of HPV [27]–[29]. In our analyses, we defined HPV 31 and 45 as closely related to and HPV 33, 52 and 58 as a distantly related to HPV 16 and/or 18.

To confirm the presence and integrity of the human DNA, beta-globin PCR analysis by PC03/04 primer set [30] was performed for all RLB assay negative specimens. Beta-globin negative specimens were excluded from our study.

The laboratory is accredited according to ČSN EN ISO 15 189 and participates regularly in external control of quality programs organized by INSTAND (Germany) and Mendel Center for Biomedical Sciences (Cyprus). Furthermore, the laboratory participated twice in WHO HPV LabNet Proficiency Study of HPV DNA Typing organized by the WHO HPV Global Reference Laboratory [31].

### HPV sequencing

To determine the type of HPV in the specimens positive by RLB only on the agarose gel but not by RLB hybridization, the remaining aliquots of PCR amplicons were used for nucleotide sequencing. The 150 bp products were cut out of the 2% NuSieve GTG agarose gel (BMA, Rockland, ME), purified using the MinElute™Gel Extraction Kit (Qiagen, Hilden, Germany) and directly sequenced using the BigDye® Terminator v1.1 Cycle Sequencing Kit (Applied Biosystems, Warrington, UK). The sequence analysis was performed on the ABI PRISM 310 genetic analyzer (Applied Biosystems) and the sequences were analyzed by Chromas software and evaluated by BLAST software (http://www.ncbi.nlm.nih.gov/BLAST/).

### Statistical analyses

Multiply infected samples were those in which two or more HPV types had been detected. Such samples were counted as positive for one type of HPV and also included among positives for the others. Type-specific HPV prevalence rates are expressed as percentages of all cases tested for HPV, and thus represent the HPV prevalence in either single or multiple infections. The differences in the mean age were assessed by a one-way analysis of variance (ANOVA) test. For contingency tables, the standard chi-square test and the Fisher exact test were used. The prevalence ratios in SCC in comparison to CIN2, 3 and CIN1 with 95% confidence intervals (CI) were determined using GraphPad InStat (version 3.00) (GraphPad Software, San Diego, CA). All tests were two sided and the significance level was p = 0.05. For assessing the possible impact of HPV vaccines on the prevention of HPV-associated cancer, we estimated the number of cervical cancer cases attributed to 8 HPV types most prevalent in the Czech Republic. We used the numbers of incident cases of cervical, vulvar and anal cancers in the Czech Republic published in 2010 [15] and type-specific HPV distribution derived from this study. A woman with multiple infections was assigned in proportional fractions to each genotype but counted only once [32].

## Results

### HPV genotyping of carcinomas

Altogether, 157 carcinoma samples were available for HPV DNA testing. Patients with SCC were significantly younger (P<0.001) than those with other types of carcinomas (see materials and methods). One hundred and eighteen (75%) carcinomas samples were HPV DNA positive. The presence of HR HPV was detected in 95% (82/86) of SCC samples, 35% (17/49) of VC samples and 82% (18/22) of AC samples. One vulvar carcinoma sample was only infected with a LR HPV genotype only (HPV 42). No LR types as a single infection were found in carcinoma samples from other anatomical locations (Table 1). Multiple infection (coinfection with two or more HPV types) was only found in 20% of SCC (17/86). Coinfection with HPV 16 and 18 was the most commmon of multiple infections (5/17). HPV 16 coinfection with HR HPV types other than HPV18 was also often observed (11/17).

**Table 1:** HPV prevalence in carcinomas of different anatomical locations.

	**Diagnosis**			
	**SCC**	**VC**	**AC**	**Total**
**Sample N**	86	49	22	**157**
	**Prevalence [%]**			
**HPV +**	95.3	36.7	81.8	**75.2**
**Single HPV**	75.6	36.7	81.8	**89.2**
**Multiple HPV**	19.8	0.0	0.0	**10.8**
**Any HR type**	95.3	34.7	81.8	**74.5**
**16**	73.3	24.5	81.8	**59.2**
**18**	8.1	0.0	0.0	**4.5**
**31**	7.0	0.0	0.0	**3.8**
**33**	10.5	8.2	0.0	**8.3**
**39**	1.2	0.0	0.0	**0.6**
**45**	9.3	2.0	0.0	**5.7**
**52**	1.2	0.0	0.0	**0.6**
**53**	1.2	0.0	0.0	**0.6**
**56**	2.3	0.0	0.0	**1.3**
**58**	3.5	0.0	0.0	**1.9**
**73**	1.2	0.0	0.0	**0.6**
**16/18** #	75.6	24.5	81.8	**60.5**
**31/45** *	8.1	2.0	0.0	**5.1**
**33/52/58** **	8.1	8.2	0.0	**7.0**
**Any LR type**	0.0	2.0	0.0	**0.6**
**42**	0.0	2.0	0.0	**0.6**
**6/11** ***	0.0	0.0	0.0	**0.0**

SCC = squamous cell cervical carcinoma, VC = vulvar carcinoma, AC = squamous cell anal carcinoma.

# samples HPV 16 and/or 18 positive.

*samples which do not contain HPV 16 and/or 18.

**samples which do not contain HPV 16 and/or 18 and/or 31 and/or 45.

***samples which do not contain HPV 16 and/or 18.

Overall, we detected 9 HR (HPV 16, 18, 31, 33, 39, 45, 52, 56, 58) 2 pHR (HPV 53, 73) and 1 LR (HPV 42) HPV types in different types of carcinomas, of which 11 different HPV genotypes were found in SCC samples while the spectrum of HR HPV types in other types of carcinomas was much narrower. Only HR HPV types 16, 33 and 45 were found as a single infection. HPV 16 was the most prevalent type in cervical 73% (63/86), vulvar 25% (12/49) and anal 82% (18/22) carcinomas, followed by HPV 33, 45, 18, and 31 in descending order.

HPV vaccinal types (HPV 16 and/or 18) were detected as a single infection in 50% (79/157) of tumors and as a coinfection with other HR HPV types in additional 10% (16/157) of samples. Altogether, 61% (95/157) of analyzed malignant tumors contained one or both vaccinal types. The presence HPV types either closely or distantly related to the vaccinal ones, i.e. HPV 31 and 45 and HPV 33, 52, and 58, respectively, was detected in additional 5% (8/157) and 11% (11/157) of carcinoma specimens, respectively. Sixty-five (76%) of 86 cervical cancer samples contained HPV 16 and/or 18 as a single or multiple infection. The presence of HPV types either closely or distantly related to the vaccinal ones, i.e. HPV 31 and 45 and HPV 33, 52, and 58, was detected in additional 8% (7/86) and 8% (7/86) of SCC samples, respectively.

### HPV genotyping of precancerous lesions

A total of 695 precancerous lesion samples were available for our analyses: 338 from CIN1 cases, 111 from CIN2 and 200 from CIN3 cases and 46 from VIN cases. Median age of women with cervical lesions was substantially and statistically significantly lower (P<0.0001) compared to that of patients with VIN (see study design). Overall, the prevalence of HPV DNA was 76% (528/695). HPV infection was detected in 62% (209/338) of CIN1 samples, 77% (85/111) of CIN2 and 94% (188/200) of CIN3 samples and 100% (46/46) of VIN samples (Table 2). Among the HPV-positive samples, 4% (19/528) were infected with LR HPV types only.

**Table 2:** HPV prevalence in precancerous lesions of different anatomical locations.

	Diagnosis				
	CIN1 (%)	CIN2 (%)	CIN3 (%)	VIN (%)	Total (%)
**Sample N**	338	111	200	46	695
**HPV +**	61.8	76.6	94.0	100.0	76.0
**Single HPV**	42.0	55.9	72.0	87.0	56.0
**Multiple HPV**	19.8	20.7	52.0	13.0	28.8
**Any HR type**	57.4	73.9	93.5	93.5	72.5
**16**	28.4	41.4	67.5	71.7	44.6
**18**	5.3	4.5	5.5	4.3	5.2
**26**	0.3	0.9	0	0	0.3
**31**	8.6	8.1	14.5	0	9.4
**33**	6.8	11.7	14.0	17.4	10.4
**35**	3.0	2.7	1.0	0	2.2
**39**	0.3	1.8	0	0	0.4
**45**	4.7	6.3	3.0	4.3	4.5
**51**	3.8	3.6	1.5	0	2.9
**52**	2.1	5.4	2.0	0	2.4
**53**	0	0.9	0	0	0.1
**56**	3.6	3.6	3.0	2.2	3.3
**58**	4.4	2.7	5.5	0	4.2
**59**	0.9	0	0	2.2	0.6
**66**	1.8	1.8	1.5	0	1.6
**68**	0.6	0	0	0	0.3
**82**	0	0	2.0	0	0.6
**Undetermined**	0.3	0.9	0	0	0.3
**55**	0	0.9	0	0	0.1
**71**	0.3	0	0	0	0.1
**16/18** #	32.5	43.2	70.5	71.7	43.0
**31/45** *	9.2	10.8	7.0	4.3	8.3
**33/52/58** **	8.9	14.4	12.5	17.4	11.4
**Any LR type**	12.4	4.5	4.0	10.9	8.6
**6**	4.1	1.8	0.5	6.5	2.9
**11**	2.4	0	0.5	2.2	1.4
**40**	0.3	0	0	0	0.1
**42**	2.1	0	1.5	0	1.4
**43**	0.6	0.9	0	2.2	0.6
**54**	0.9	0.9	0.5	0	0.7
**70**	1.5	0.9	0.5	0	1.0
**81**	1.2	0.9	0.5	0	0.9
**89**	0.9	0	0	0	0.4
**6/11** ***	5.3	0.9	0.5	8.7	2.9

CIN1 = cervical intraepithelial neoplasia grade 1, CIN2/3 =  cervical intraepithelial neoplasia grade 2 and 3, VIN = vulvar intraepithelial neoplasia.

# samples HPV 16 and/or 18 positive.

*samples which do not contain HPV 16 and/or 18.

**samples which do not contain HPV 16 and/or 18 and/or 31 and/or 45.

***samples which do not contain HPV 16 and/or 18.

In comparison to carcinomas, precancerous lesions contained a larger variety of HPV types. As shown in Table 2, altogether 28 different HPV genotypes were detected: 13 were HR (HPV 16, 18, 31, 33, 35, 39, 45, 51, 52, 56, 58, 59, 68), 4 pHR (HPV 26, 53, 66, 82) and 9 LR (HPV 6, 11, 40, 42, 43, 54, 70, , 81, 89) and 2 undetermined (HPV 55, 71). Most of these types were present in cervical lesions. The VIN samples were infected with 6 different HR (HPV 16, 18, 33, 45, 56, 59) and 3 LR (HPV 6, 11, 43) HPV types. Similar to carcinomas, HPV 16 was the most prevalent type in all types of precancerous lesions followed by HPV 33, 31, 18, and 45. HPV 16 was observed in 28% (96/338) of CIN1 samples, 41% (46/111) of CIN2 and 68% (135/200) of CIN3 samples and 72% (33/46) of VIN samples.

More than a half of samples contained a single HPV type (56%). Multiple infection was found most commonly in cervical precancerous lesion samples: in 20% (66/338) of CIN1, 21% (23/111) of CIN2 and 22% (44/200) of CIN3 samples. Most multiple infections were coinfections with two or three HPV types. Coinfection with four HPV genotypes was only found in six cervical lesion samples.

The vaccinal types HPV 16 and/or 18 were present in 34% (239/695) of precancerous lesion samples as a single infection, in 1% (8/695) as a multiple infection (combined HPV16/18 infection), and in 13% (87/695) in combination with other HPV types. The presence of the types either closely or distantly related types to the vaccinal ones: HPV 31 and 45 and HPV 33, 52, and 58, respectively, was detected in 9% (59/695) and 11% (79/695) of samples, respectively.

Altogether, 33% (110/338), 43% (48/111) and 71% (141/200) of CIN1, CIN2 and CIN3 samples, respectively, were positive for HPV 16 and/or 18. Closely related types HPV 31 and 45 were present in 9% (31/338),11% (12/111) and 7% (14/200) of samples, respectively, and distantly related types HPV 33, 52, and 58 in 9% (30/338), 14% (16/) and 13% (25/100) of samples, respectively. LR HPV types were detected in 9% of precancerous lesion samples. HPV 6 and/or 11 were present as either a single or multiple infection in 3% of all precancerous lesion samples, more precisely in 5% (18/338) of CIN1 samples, 1% (1/111) of CIN2 and 1% (1/200) of CIN 3 samples and 9% (4/46) of VIN samples.

### HPV genotyping of condylomata acuminata

HR HPV types as a single infection were only detected in 4% (2/54) of VCA samples and as a multiple infection together with LR types in additional 15% (8/54) of VCA samples, while ACA samples did not contain any HR HPV type, with a single sample being positive for two LR types. In VCA samples, 11 different HPV types were detected: 4 were HR (HPV 16, 33, 45, 51), 2 pHR (HPV 26, 73) and 5 LR (HPV 6, 11, 42, 84, 81) HPV types (Table 3).

**Table 3:** HPV prevalence in condyloma acuminata of different anatomical locations.

	**Diagnosis**		
	**VCA**	**ACA**	**Total**
**Sample N**	54	10	**64**
	**Prevalence [%]**		
**HPV +**	94.4	70.7	**90.6**
**Single HPV**	75.9	60.0	**73.4**
**Multiple HPV**	18.5	10.0	**17.2**
**Any HR type**	18.5	0	**15.6**
**16**	1.9	0	**1.6**
**26**	3.7	0	**3.1**
**33**	3.7	0	**3.1**
**45**	1.9	0	**1.6**
**51**	1.9	0	**1.6**
**73**	5.6	0	**4.7**
**16/18** #	1.9	0	**1.6**
**31/45** *	1.9	0	**1.6**
**33/52/58** **	3.7	0	**3.1**
**Any LR type**	90.7	100.0	**89.1**
**6**	75.9	50.0	**71.9**
**11**	18.5	30.0	**20.3**
**42**	3.7	0	**3.1**
**84**	1.9	0	**1.6**
**81**	1.9	0	**1.6**
**6/11** ***	87.0	70.0	**84.4**

VCA  =  vulvar condyloma acuminatum, ACA  =  anal condyloma acuminatum.

# samples HPV 16 and/or 18 positive.

*samples which do not contain HPV 16 and/or 18.

**samples which do not contain HPV 16 and/or 18 and/or 31 and/or 45.

***samples which do not contain HPV 16 and/or 18.

Altogether, vaccinal HPV types 6 and/or 11 were present in 84% (54/64) of condyloma acuminatum samples from different anatomical locations, in 87% (47/54) of VCA samples, and 70% (7/10) of ACA samples.

### Comparison of HPV prevalence in SCC with CIN1, CIN2 and CIN3 cases

The overall prevalence of HPV was higher in SCC (95%) in comparison to CIN1 (62%) (SCC:CIN1 ratio 1.5, 95% CI 1.4–1.7), CIN2 (77%) (SCC:CIN2 ratio 1.2, 95% CI 1.1–1.4) but not in CIN3 (94%) (SCC:CIN3 ratio 1.0, 95% CI 1.0–1.1) cases. Similar results were obtained for any HR HPV type detected and for seven HR HPV types most prevalent in SCC samples (Table 4). For each of the seven HPV types, the SCC:CIN1, SCC:CIN2 and SCC:CIN3 ratios were also calculated. The respective ratios were 2.6, 1.8 and 1.1 for HPV 16, 1.5, 1.8 and 1.5 for HPV 18 and 2.0, 1.5 and 3.1 for HPV 45. For HPV types closely related (HPV 31, 33, 56), or distantly related (HPV 58) the SCC:CIN3 ratios were 0.5 to 0.8, respectively (Table 4).

**Table 4:** Comparison of overall and type-specific HPV prevalence between CIN1 and SCC, CIN2 and SCC, and CIN3 and SCC cases.

	CIN1:SCC			CIN2:SCC			CIN3:SCC		
HPV type	PREVALENCE RATIO			PREVALENCE RATIO			PREVALENCE RATIO		
	RR	95%CI	P	RR	95%CI	P	RR	95%CI	P
All**	1.5	1.40–1.70	<0.0001	1.2	1.11–1.39	0.0002	1.0	0.96–1.08	0.784
Any HR***	1.7	1.50–1.84	<0.0001	1.3	1.13–1.43	<0.0001	1.0	0.96–1.08	0.786
7 HR****	1.8	1.61–2.05	<0.0001	1.7	1.44–2.06	<0.0001	1.0	0.95–1.09	0.814
16	2.6	2.09–3.19	<0.0001	1.8	1.37–2.28	<0.0001	1.1	0.92–1.27	0.402
18	1.5	0.66–3.54	0.312	1.8	0.59–5.50	0.372	1.5	0.59–3.69	0.430
31	0.8	0.35–1.90	0.826	0.9	0.32–2.33	1.000	0.5	0.22–1.21	0.157
33	1.5	0.74–3.20	0.256	0.9	0.40–1.99	0.824	0.8	0.37–1.52	0.450
45	2.0	0.87–4.44	0.117	1.5	0.56–3.91	0.589	3.1	1.11–8.67	0.034
56	0.7	0.15–2.87	0.745	0.7	0.12–3.44	0.698	0.8	0.16–3.77	1.000
58	0.8	0.23–2.66	1.000	1.3	0.27–6.24	1.000	0.6	0.18–2.22	0.564

95%CI = 95% confidence interval, RR =  relative risk, P =  probability.

**all HPV types detected (LR and HR).

***all HR HPV types detected.

****HR HPV types 16, 18, 31, 33, 45, 56, and 58.

### Cervical, vulvar and anal cancers associated with specific HPV types

**Figure 1:**
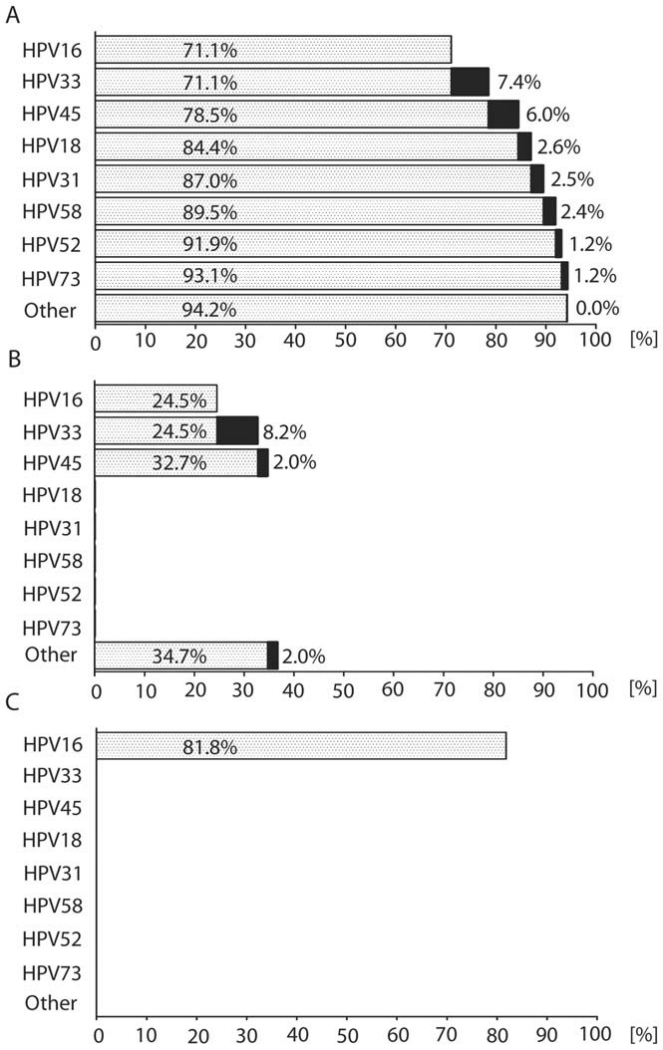
Cumulative percentages of cancer cases of women and men in the Czech Republic. Cumulative percentages of cervical (A), vulvar (B) and anal (C) cancer cases in women and men occurring every year in the Czech Republic that are attributed to eight most prevalent HPV types (990, 189 and 121 incident cancer cases, respectively). (Sdapted from Munoz, 2004)[46].

In the Czech Republic 1300 cervical, vulvar and anal incident cancer cases occur, more precisely 990 cervical cancer cases, 189 vulvar cancer cases, and 121 anal cancer cases [15]. We estimated the number of cases that can be attributed to the 8 most prevalent HPV types from this study (HPV 16, 18, 31, 33, 45, 52, 58, and 73). The proportion of cancer cases attributed to the 8 most prevalent HPV types was 74.5% which corresponds to 961 cancer cases. The particular rates were 94.2% for SCC, 34.7% for VC and 81.8% for AC. The eight most prevalent HPV types account for 932 SCC, 66 VC, and 99 AC cases in the Czech republic (Figure 1).

## Discussion

In this study we provide the largest summary data on the type-specific HPV type specific prevalence in the population of Czech women and men with diseases of the anogenital tract associated with HPV infection. Importantly, the prevalence rates of the vaccinal HPV types as well as those of HPV types which have shown a partial cross protection in clinical trials [33]–[36] are reported. The results of this study allow estimating potential benefit that can be achieved by the implementation of routine vaccination in the Czech Republic. Furthermore, these results will be used as inputs for models for estimating the impact of different strategies for the prevention of HPV-associated diseases.

In this study we analyzed 157 squamous cell carcinoma samples, 695 precancerous lesion samples and 64 condylomata acuminata samples from different anatomical locations. A very sensitive method was used, which is based on the amplification of a short DNA fragment of HPV L1 ORF and allows detection of multiple HPV infections [26]. This method is also recommended for HPV detection in primary screening by the European Guidelines for Quality Assurance in Cervical Cancer Screening [37]. Furthermore, the classification of all samples analyzed in this study, except for 14% of CIN1 samples, was confirmed by histology.

The data on HPV prevalence in precancerous cervical lesions and invasive cervical cancer cases in the Czech Republic were evaluated previously on another set of specimens [21]. The HPV prevalence was much lower in the previous study compared to the present one (53% vs. 62% in CIN1 samples, 58% vs. 88% in CIN2/3 samples and 74% vs. 95% SCC samples) as were the numbers of different HPV types (16 HR, 5 LR und 1 undetermined HPV types vs. 17 HR, 9 LR HPV types and 2 undetermined). Even though yielding very important results, our previous study had some limitations. Relatively small numbers of precancerous cervical lesion samples and cervical carcinoma samples were analyzed (87 CIN1, 88 CIN2+, and 49 SCC samples). The PCR method then used for HPV detection, in wide use at that time, has shown limited sensitivity in comparison to other newly introduced ones [38]. Finally, the severity of precancerous lesions was not confirmed for all cases by histology in our previous study. Therefore, the discrepancy in results between our two studies can be most likely attributed to the above-mentioned factors.

The published data on the type-specific HPV prevalence in patients with HPV-associated diseases in the Central and East European countries are scarce and most studies have analyzed only very small numbers of specimens. While more studies from Central and East Europe on HPV prevalence in CC were published and included in the meta-analyses [9], the HPV prevalence in CIN1 samples was only reported for the Czech republic [7] and that in CIN2+ samples for the Czech Republic and Hungary [9], [13]. Recently few additional studies from the Central and East European region have been published [17], [39], [40], but only that of Bardin et al. [17] reported on a larger number of SCC patients. Two meta-analyses [9], [13] have concordantly shown an overall rate of 85% of HPV positivity of SCC samples in Europe. The prevalence rates ranged from 53 to 100% and that for the Czech Republic is 95%, as determined in this study. The most prevalent HPV types in SCC in Europe are, in descending order, HPV 16, HPV 18, and HPV 45. In our study, the second most common type was HPV 33, followed by HPV 45 and HPV 18. Since only squamous cell carcinoma samples were included in our study, the reported HPV type prevalence data is in agreement with the results of the meta-analysis of Smith et al. who has shown variation in type-specific HPV prevalence between squamose cell carcinomas and adenocarcinomas [9]. In accordance with Smiths data for the Europe region, HPV 56 is more common in the Czech Republic than HPV 52 which is more prevalent in SCC in other regions of the world [41]. Despite the fact that HPV 35 was the sixth most prevalent type in SCC in the recently published study by de Sanjose et al. [41], we didńt detect this HPV type in our cohort of SCC patients. In the previous meta-analyses the majority of studies from Europe didńt find HPV 35 in SCC as well, regardless of method used for HPV detection [9], [13]. Since in the WHO proficiency study [31] both methods; SPF-10 PCR used in the recent study and GP5+/6+ RLB used in our study, proofed to be very sensitive for the detection of HPV 35, we conclude that discrepant findings can be explained only by the differences in the number of cases studied (86 SCC specimens vs. 2093 SCC specimens from Europe) [41].

The type-specific HPV prevalence in CIN2+ samples found in our study, is the same as the data reported for Europe (88%), with the exception of HPV 16 and/or 18 (61 vs. 52%) [9]. The prevalence rates of other HPV types detected were similar to those observed in Europe, apart from HPV 73 that was not recovered from CIN2+ cases in the Czech Republic, most likely as a result of the use of a less sensitive assay for the detection of HPV 73 [42].

In comparison to the summary data for Europe as published by Clifford et al. [7], the type-specific HPV prevalence in CIN1 cases in our study was quite different. We detected about a one third higher prevalence of HPV 16 (28 vs. 19%), but much lower prevalence rates of HPV 59 (1% vs. 3%), HPV 39 (0.3% vs. 3%), HPV 66 (2% vs. 6%), HPV 52 (2% vs. 5.4%), and HPV 53 (0% vs. 3.7%). The spectrum of HPV types present in CIN1 cases is much wider in comparison to CIN2+ and SCC, with the low prevalent types being more common. Our group has previously reported that the detection of low prevalent types can vary greatly between different assays and that RLB with GP5+/6+ primers has lower sensitivity for HPV 52, 53, and 59 [42]. This could explain some of the discrepant findings.

The present study has shown a significantly higher prevalence of any HPV type, as well as of HR HPV type among SCC cases in comparison to CIN1 (p<0.0001 for both)and CIN2 cases (p = 0.0002 and p<0.0001, respectively) (Table 4). The prevalence was also higher for the seven HPV types most prevalent in SCC. HPV 16 was significantly more prevalent in SCC cases in comparison both to CIN1 (p<0.0001) and CIN2 (p<0.0001) cases but not to CIN3 cases (p = 0.402). Prevalence ratios above one were recorded for HPV 18, 45 and 58, while for other HPV types, the ratios ranged between 0.5 and 0.9, but except for HPV 45 (p = 0.034) in CIN3 in comparison to SCC, the differences were not statistically significant. Since our data are comparable to the ratios reported by Clifford et al. [6] for a large number of cases, we conclude that the lack of statistical significance for HPV types other than HPV 16 is due to the small numbers of subjects positive for HPV types other than HPV 16.

A meta-analysis of HPV prevalence studies in precancerous lesions and vulvar and anal carcinomas [11], [12] included our previously published data from the Central and Eastern European region and those from Poland and Austria. Recently studies of Kowalewska et al. [43] and Garland et al. [44] have reported on HPV prevalence in vulvar cancer in Poland and Austria. In our study, the HPV prevalence in VIN cases was 100%. Most other studies which have reported comparably high prevalence rates only included patients with VIN 3. We have detected HPV in all VIN samples, including VIN 1 and 2. The most prevalent type was HPV 16, followed by HPV 33, 18, and 45. This finding is in agreement with the summary data reported by de Vuyst et al. [12], except the prevalence rates of HPV 33 and 45 were higher in this study. The lower average rates in the meta-analysis are due much lower prevalence of HPV 33 in many studies and almost no detection of HPV 45 in VIN.

The overall prevalence of HPV as well as HPV type distribution in VC is in our study comparable to other studies. Nowadays it is widely accepted that about 40% of VC cases can be etiologically linked to HPV.

Importantly, focused on the prevalence of both vaccinal and cross-reactive HPV types, this study revealed that altogether 43% of precancerous lesions of the cervix and vulva (33% of CIN1, 43% of CIN2, 71% of CIN3 and 72% of VIN) are caused by HPV 16 and/or 18 and additional 20% by HPV types related to the vaccinal ones (HPV 31, 45, 33, 52, 58). Therefore, a substantial number of precancerous lesions can be considered preventable by prophylactic vaccination in the Czech Republic. The vaccinal LR HPV types HPV 6 and/or 11 were detected in 5% of CIN1, as few as 1% of CIN2 and 0.5% CIN3 and 9% of VIN cases.

The overall prevalence of HPV 16 and/or 18 among the analyzed cancer cases was 61% and that of the closely or distantly related types was 12%. The lowest prevalence of HPV 16/18 was observed in VC cases. Based on our data, the development of vulvar cancer can be prevented in about half of cases, thus reducing the need for mutilating surgery that dramatically reduces quality of life for patients.

The rate of SCC cases attributable to HPV 16/18 infection in the Czech population is 76%. Even higher is the involvement of HPV 16/18 in AC cases (82%). In view of cross-protective effect of the available vaccines, we can expect the potential benefit from vaccination against HPV in preventing SCC to be as high as 92% for the Czech population.

Finally, specimens with the histologically confirmed presence of condylomata acuminata were analyzed. Even though only a limited number of samples were available, the information is very important for the planning of the preventive strategies. We have shown that 89% of these lesions are infected by LR HPV types, with vaccinal types HPV 6/11 being present in 84% of them. These data should be taken into account when considering population-based prophylactic vaccination against HPV.

In conclusion, our study reports on the type-specific prevalence of HPV in benign, premalignant and malignant lesions of the anogenital tract in women and man. The prevalence and spectrum of HPV types detected in the Czech Republic are comparable to the data reported for European countries. The observed differences can be mostly attributed to the variation in the methods used for HPV detection. The proportion of patients infected with vaccinal and closely or distantly related HPV types is much higher than originally proposed. Approximately 952 of 1300 incident cancer cases (CC, VC and AC) and 921 of 990 CC cases can be attributed to these HPV types in the Czech Republic. Furthermore, it has been shown that the implementation of routine vaccination not only resulted in decrease in incidence of atypical cervical cytology and precancerous cervical lesions but also in the reduced need for colposcopy and invasive treatment procedures [45]. Therefore, we strongly advocate a rapid implementation of routine HPV vaccination in the Czech Republic which can significantly reduce the burden of HPV-associated diseases as well as the national healthcare expenditures.
